# Routine Cerebrospinal Fluid Cytology Reveals Unique Inclusions in Macrophages During Treatment With Nusinersen

**DOI:** 10.3389/fneur.2019.00735

**Published:** 2019-07-11

**Authors:** Stefan Gingele, Martin W. Hümmert, Sascha Alvermann, Konstantin F. Jendretzky, Lena Bönig, Marina Brieskorn, Philipp Schwenkenbecher, Kurt-Wolfram Sühs, Lars H. Müschen, Alma Osmanovic, Olivia Schreiber-Katz, Martin Stangel, Susanne Petri, Thomas Skripuletz

**Affiliations:** Department of Neurology, Hannover Medical School, Hanover, Germany

**Keywords:** nusinersen, spinal muscular atrophy (SMA), cerebrospinal fluid (CSF), cytology, macrophage, monocyte, lymphocyte

## Abstract

**Background:** Spinal muscular atrophy (SMA) is an autosomal recessive neurodegenerative disorder characterized by degeneration of spinal motor neurons leading to muscular weakness. The antisense oligonucleotide nusinersen was approved for the treatment of patients with 5q-associated SMA. Treatment must be repeatedly administered intrathecally by lumbar puncture. So far, data regarding cerebrospinal fluid (CSF) parameters are sparse and examinations of CSF cytology during nusinersen treatment are completely missing.

**Methods:** 87 CSF samples from 19 adult SMA patients who underwent repeated lumbar punctures for intrathecal injections of nusinersen were investigated. CSF specimens were quantitatively assessed regarding leukocyte subpopulations by routine cytology after Pappenheim staining. A control group with 38 CSF samples from 10 patients with repeated lumbar punctures due to other diseases was used.

**Results:** Treatment with nusinersen did not result in persistent inflammatory cellular changes or a relevant shift of leukocyte subpopulations in the CSF. During nusinersen therapy unique macrophages with numerous sharply defined purple and granular inclusions were detected in all patients. These macrophages were not found in CSF of patients with other diseases who underwent repeated lumbar punctures.

**Discussion:** Routine CSF cytology performed by experienced personnel represents an important and feasible tool for safety monitoring during treatment with intrathecally administered therapeutics. Analysis of leukocyte subpopulations did not raise safety concerns during nusinersen therapy. The potential significance of the unique phagocytic cells for disease course and treatment response needs to be further elucidated in the future.

## Introduction

Spinal muscular atrophy (SMA) is an autosomal recessive neurodegenerative disorder characterized by degeneration of lower motor neurons in the spinal cord with subsequent progressive muscular atrophy and weakness (1). The disease is caused by disruption of the survival motor neuron 1 (*SMN1*) gene on chromosome 5q13 (2). The antisense oligonucleotide nusinersen modifies the pre-mRNA splicing of the survival motor neuron gene *SMN2* which leads to increased production of full-length SMN protein, thereby compensating for the genetic defect in the *SMN1* gene and improving motor function in patients with different SMA phenotypes (3–5). Nusinersen (Spinraza®) was approved by the FDA in the USA in 2016 and by the EMA in the European Union in 2017 for the treatment of patients with 5q-associated SMA. Since nusinersen cannot pass the blood–brain barrier it has to be repeatedly administered by intrathecal injection via lumbar puncture on days 0, 14, 28, and 63 and subsequently every 4 months (6). During phase 1 and 2 clinical studies prior to approval of nusinersen, cerebrospinal fluid (CSF) was analyzed for safety assessments regarding total cell count, protein, glucose, inflammatory cytokines, and anti-nusinersen antibodies which revealed no safety concerns (7, 8). Apart from a recent letter reporting a decrease of elevated tau and neurofilament light chain levels in an infant with SMA (9) and the description of the macroscopic condition of the CSF after lumbar puncture in adolescent and adult SMA patients (6), data regarding CSF parameters during nusinersen therapy are sparse and investigations of CSF cytology during nusinersen therapy are completely missing. Therefore, we examined the impact of repeated intrathecal administrations of nusinersen in adult SMA patients on CSF cytology.

## Patients and Methods

### Patients

Routine CSF cytology specimens of adult patients with genetically proven 5q-SMA who were treated with nusinersen at the Department of Neurology at Hannover Medical School between November 2017 and January 2019 were analyzed. In total, we collected 87 CSF samples from 19 adult patients with SMA (8 patients with SMA type 2, 11 patients with SMA type 3) who underwent repeated lumbar punctures (ranging from three to seven) for intrathecal injections of nusinersen on days 0, 14, 28, 63, and at month 6, 10, and 14. Median age was 35 years, ranging from 19 to 64 years. Seven female and 12 male patients were included into the analysis. 38 CSF cytology samples from mostly age-matched patients who underwent repeated lumbar punctures at least twice within 6 months between 2016 and 2019 with diagnosis of idiopathic intracranial hypertension (IIH) (*n* = 8), meningitis (*n* = 1) or chronic inflammatory CNS disorder (*n* = 1) served as control group. Median age was 35 years, ranging from 28 to 61 years. This investigation was approved by the Ethics Committee of Hannover Medical School (No. 3142-2016).

### Preparation of CSF Samples

CSF was drained prior to administration of nusinersen and was subjected to standard diagnostic procedures within 1 h after lumbar puncture in the neurochemistry laboratory at the Department of Neurology as described previously (10). Briefly, cells in the CSF were manually counted using a Fuchs-Rosenthal counting chamber and a CSF cell count of > 4 cells/μl was considered as elevated. Fifteen minutes pre-centrifugation with 145 g of 2–5 ml CSF was performed to enrich the cells. Cell sediment was resuspended in 0.2 ml of cell culture medium and cytospins were prepared in a Shandon Cytospin 3 (Thermo Shandon Limited, Cheshire, UK) at 90 g for 10 min (11). After air drying CSF specimen were stained using a panoptic Pappenheim stain by performing May-Grünwald (Merck, Darmstadt, Germany) followed by Giemsa staining (Sigma-Aldrich, St. Louis, USA). To distinguish macrophages with blue or purple inclusions from siderophages (**Figure 2C**), prussian blue staining for iron was performed regularly. Dried cells were immersed in a solution of 2% potassium hexacyanoferrate and 1% hydrochloric acid (Merck, Darmstadt, Germany) followed by a counterstaining with nuclear fast red aluminum sulfate solution (Carl Roth, Karlsruhe, Germany) (12).

### Analysis of CSF Cytology Specimens

Specimens were independently examined by two raters experienced in CSF cytology who were blinded to condition and time point. In samples from SMA patients, the following leukocyte populations were assessed quantitatively: lymphocytes, plasma cells, monocytes, macrophages, erythrophages, erythrosiderophages, siderophages, neutrophils, eosinophils, and basophils. Cartilage and bone marrow cells were present in some specimens but were not analyzed. In cases where raters either disagreed on the presence or absence of any cell type or the percentage of lymphocytes and/or monocytes differed by more than 10% between the two raters' assessments, the specimen was reviewed and discussed by both raters together. After consensus was reached and data were corrected accordingly, mean values of both raters' assessments were calculated for each specimen and cell population. Specimens from repeatedly punctured patients with other diseases were assessed as a control. Prussian blue stainings were analyzed regarding iron containing siderophages. Specimens were examined using an x40 objective (Axiostar, Carl Zeiss, Jena, Germany).

### Statistical Analysis

All statistical analyses were performed using GraphPad Prism 8.0 (GraphPad Software, San Diego). Data are given as arithmetic means ± standard error of the mean (SEM).

## Results

### CSF Cytology During Nusinersen Treatment

**Figure 1:**
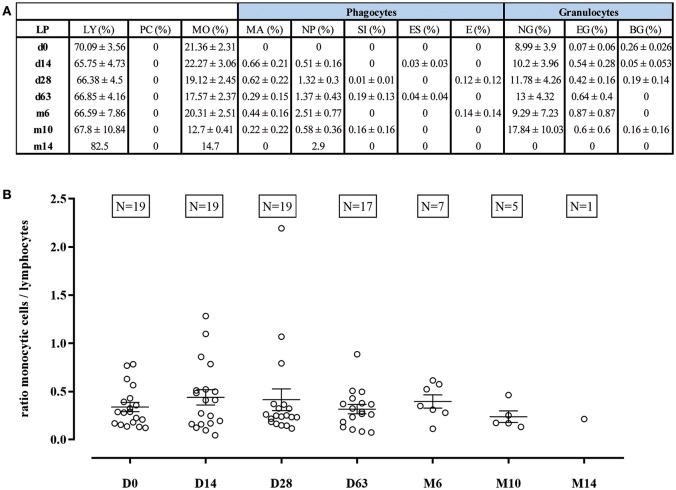
Cell distribution of leukocyte subpopulations during treatment with nusinersen. Respective cell populations are given as proportion of total leukocytes in percentage with mean ± standard error of the mean (SEM) **(A)**. Ratio between share of monocytic cells (including monocytes and different forms of macrophages termed as “phagocytes”) and lymphocytes is depicted at different time points of nusinersen therapy **(B)**. Dot plots show mean ± SEM. LP, lumbar puncture; LY, lymphocytes; PC, plasma cells; MO, monocytes; MA, macrophages; NP, nusinophages; SI, siderophages; ES, erythrosiderophages; E, erythrophages; NG, neutrophil granulocytes; EG, eosinophil granulocytes; BG, basophil granulocytes.

At the time of first lumbar puncture before treatment with nusinersen, all patients showed a normal CSF leukocyte cell count (<4 cells/μl). Evaluation of all 87 CSF samples revealed mild pleocytosis (ranging from 5 to 21 cells/μl) in 4 specimens which did not persist at the subsequent lumbar punctures (Supplemental Table 1). The proportions of the different leukocyte subpopulations showed considerable variation between the individual punctures which was predominantly attributable to admixture of peripheral blood cells to the CSF samples (Supplemental Table 1). Apart from presumably peripherally derived granulocytes, other phagocyte populations were not detected at the first lumbar puncture but were evident during repeated punctures at a small percentage (Figure 1A). In addition, there were no relevant changes in the ratio of the CSF-resident cell populations of monocytic cells (comprising monocytes and macrophages termed as “phagocytes”) and lymphocytes (Figure 1B). In addition, plasma cells were not detectable in any of the 87 CSF specimens. Taken together, quantitative analysis of leukocyte subpopulations did not provide evidence for a sustained inflammatory cellular reaction in the CSF or a relevant shift of the cellular composition of the CSF under treatment with nusinersen.

### Emergence of Unique Macrophages (“Nusinophages”)

**Figure 2:**
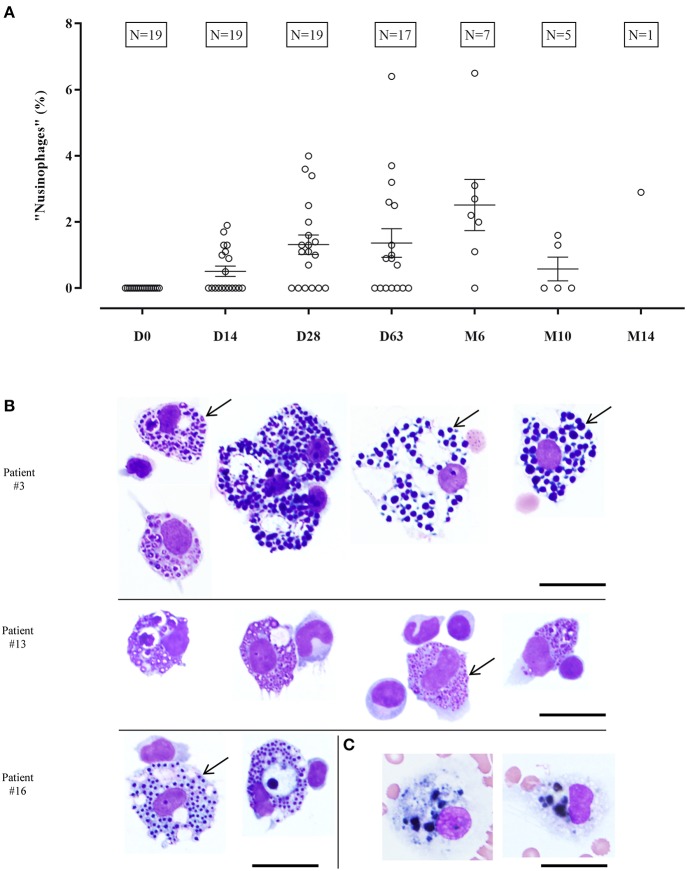
Incidence of characteristic macrophages containing numerous sharply defined blue or purple granules (“nusinophages”). The proportion of “nusinophages” at the time of the respective lumbar puncture is stated as percentage of all leukocytes **(A)**. Dot plots show mean ± SEM. Macrophages with characteristic sharply demarcated inclusions classified as “nusinophages” are exemplarily shown in CSF specimen of patients who had no relevant admixture of erythrocytes in the prior lumbar punctures **(B)**. Arrows display characteristic inclusions in different “nusinophages.” Phagocytes graded as siderophages from a CSF specimen of a patient under Nusinersen treatment after a prior lumbar puncture with >1,000 erythrocytes/μl **(C)**. Scale bar = 25 μm.

Beginning with the second lumbar puncture, macrophages with sharply defined purple and blue granular inclusions were detected whereas these cells were not present in any specimen of the first lumbar puncture before treatment with nusinersen (Figure 2A). These macrophages were found in CSF samples of every patient at least at one time point during nusinersen therapy and accounted for 0.5–6.5% of all leukocytes (Supplemental Table 1). At the time of the second lumbar puncture, no CSF specimen contained more than 2% of these macrophages and a higher percentage of up to 6.5% was found in samples from later time points. However there was no clear increase in the percentage of macrophages with inclusions over time and the two outliers with >6% of these cells at d63 and m6 as well as the small number of samples at later time points should be noted (Figure 2A).

These cells, which we termed “nusinophages” for convenience, were characterized by numerous sharply demarcated inclusions varying in size and in color between purple and blue (Figure 2B). Prussian blue staining for iron did not show siderophages in specimens in which only “nusinophages” but not siderophages had previously been detected by Pappenheim staining. In addition, 38 CSF samples of 10 patients who had undergone repeated lumbar punctures due to other diseases were investigated for the presence of “nusinophages.” No phagocytic cells resembling these macrophages with characteristic sharply defined granules seen in patients under treatment with nusinersen (“nusinophages”) were observed in these patients (Supplemental Table 2).

## Discussion

The antisense oligonucleotide nusinersen (Spinraza®) must be repeatedly administered intrathecally for the treatment of patients with 5q-associated SMA. Monitoring of potential alterations of CSF parameters and especially changes of the cellular composition of the CSF is crucial.

Here, we systematically analyzed leukocyte subpopulations in adult SMA patients treated with nusinersen. Since no evidence for sustained inflammatory cellular reactions or a relevant shift of the cellular composition in the CSF was found, cytological analysis did not reveal safety issues of nusinersen treatment in adult patients with SMA. These findings complement the extensive clinical data demonstrating safety and feasibility of nusinersen therapy (6, 13–15).

Interestingly, during the treatment with nusinersen unique macrophages with numerous sharply delineated blue and purple inclusions were detected in all patients. These cells were not found in patients who had undergone repeated lumbar punctures due to other diseases and we are not aware of the existence of similar CSF macrophages in other conditions. However, different intervals between the lumbar punctures of SMA patients and control group patients represent a limitation of this comparison. Since stainings for oligonucleotides in an infant treated with nusinersen indicated the presence of nusinersen in neuronal and non-neuronal cells in brain and peripheral tissue (8) we speculate, that the inclusions found in the macrophages (colloquially termed “nusinophages”) might also contain nusinersen. However, the origin and significance of these remarkable phagocytes remains unclear.

In summary, cells with unique inclusions were detected in the CSF of patients after repeated administration of nusinersen. Further investigations are needed to clarify whether these inclusions might contain nusinersen and if these cells may influence the response to therapy. We suggest that analysis of CSF parameters should supplement clinical measures of safety and therapy response during treatment with intrathecally delivered drugs.

## Data Availability

All datasets generated for this study are included in the manuscript and/or the Supplementary Files.

## Ethics Statement

This study was carried out in accordance with the recommendations of the institutional ethics committee of Hannover Medical School with written informed consent from all subjects (No. 3142-2016). All subjects gave written informed consent in accordance with the Declaration of Helsinki. The protocol was approved by the institutional ethics committee of Hannover Medical School.

## Author Contributions

SG, MH, SA, LB, and TS evaluated the specimens. SG, MH, SA, KJ, PS, K-WS, LM, AO, OS-K, MB, MS, SP, and TS analyzed the data. SG, MH, SA, KJ, MS, SP, and TS drafted and wrote the manuscript. All authors contributed to manuscript revision, read, and approved the submitted version.

### Conflict of Interest Statement

The authors declare that the research was conducted in the absence of any commercial or financial relationships that could be construed as a potential conflict of interest.
